# Factors affecting compliance with the measles vaccination schedule in a Brazilian city

**DOI:** 10.1590/S1516-31802008000300006

**Published:** 2008-05-01

**Authors:** Patricia Logullo, Heráclito Barbosa de Carvalho, Renata Saconi, Eduardo Massad

**Keywords:** Measles vaccine, Communications media, Guideline adherence, Communication, Compliance, Vacina contra sarampo, Meios de comunicação, Fidelidade a diretrizes, Comunicação em saúde, Complacência (medida de distensibilidade)

## Abstract

**CONTEXT AND OBJECTIVE:**

The success of vaccination campaigns depends on the degree of adherence to immunization initiatives and schedules. Risk factors associated with children's failure to receive the measles vaccine at the correct age were studied in the city of São Paulo, Brazil.

**DESIGN AND SETTING:**

Case-control and exploratory study, in the metropolitan area of São Paulo.

**METHODS:**

The caregivers of 122 children were interviewed regarding their perceptions and understanding about the measles vaccination and the disease.

**RESULTS:**

The results showed that age, region of residence, marital status and education level were unrelated to taking measles vaccines adequately. Most individuals remembered being informed about the last annual vaccination campaign by television, but no communication channel was significantly associated with vaccination status. The answers to questions about knowledge of the disease or the vaccine, when analyzed alone, were not associated with taking measles vaccinations at the time indicated by health agencies. The results showed that, when parents felt sorry for their children who were going to receive shots, they delayed the vaccination. Most of the children did not take the measles vaccination on the exactly recommended date, but delayed or anticipated the shots.

**CONCLUSION:**

It is clear that there is no compliance with the government's recommended measles vaccination schedule (i.e. first dose at nine and second at 15 months of age, as recommended in 1999 and 2000). Feeling sorry for the children receiving shots can delay vaccination taking.

## RESUMO

**CONTEXTO E OBJETIVO:**

O sucesso das campanhas de vacinação depende da adesão às iniciativas e ao calendário de imunização. Fatores de risco associados com não tomar a vacina contra o sarampo na idade recomendada foram estudados na cidade de São Paulo.

**TIPO DE ESTUDO E LOCAL:**

Estudo caso-controle e exploratório, realizado na área metropolitana de São Paulo.

**MÉTODO:**

Cuidadores de 122 crianças foram entrevistados sobre sua percepção e compreensão sobre a vacina contra o sarampo e sobre a doença.

**RESULTADOS:**

Os resultados mostraram que idade, região de residência, status marital ou nível educacional não se relacionaram com a tomada adequada das vacinas contra o sarampo. A maioria dos indivíduos lembrou-se de ter sido informada sobre a última campanha nacional de vacinação na televisão, mas nenhum canal de comunicação se associou significativamente com o status vacinal. As respostas às questões sobre conhecimentos a respeito da doença e da vacina, quando analisadas em separado, não se associaram à tomada das vacinas nas datas indicadas pelas instituições de saúde. Os resultados mostraram que quando os pais sentiam dó ao verem os filhos tomando as injeções, eles atrasavam a vacinação por pelo menos 20 dias. A maioria das crianças não toma a vacina contra o sarampo exatamente no dia recomendado, mas atrasa ou antecipa as injeções.

**CONCLUSÃO:**

Esta claro que não há aderência ao calendário governamental recomendado de vacinação contra o sarampo (i.e. primeira dose aos 9 e segunda dose aos 15 meses de idade, como recomendado em 1999 e 2000). Sentir pena de ver a criança recebendo uma injeção pode atrasar a tomada da vacina.

## INTRODUCTION

### Why do children not get vaccinated?

Many studies all over the world have investigated why people do not vaccinate their children.^1–8^ A review by Nigenda-Lópes covering 40 years showed a variety of reasons: cultural, such as “vaccinations are not important”; psychological, such as “vaccines are unsafe”; social or socioeconomic, such as parents’ educational, marital or economic status; structural, such as in relation to vaccine distribution; and transmission-related, i.e. failure to transmit enough information about the diseases and vaccines available.^3^ These reasons seem to apply to different communities.^7,8^

Two different studies in the city of São Paulo, Brazil, have shown that mothers interviewed just before having their children vaccinated knew a great deal about the reactions caused by the shots (such as fever or rash) but virtually nothing about the diseases against which the vaccines protected. They had little notion of their own children's vaccination status and could not understand terms written on the vaccination card.^9,10^ Another study showed that the vaccination schemes of the children of more than half of the parents interviewed in a slum in São Paulo were incomplete. Nevertheless, these parents considered their children's vaccination schemes to be correct based on “information given by health staff”.^11^

The present study was conducted to try to find out the reasons why people were not taking their children to be vaccinated against measles, up to the year 2000. It considered structural, socioeconomic, psychological, cultural and communicational factors. It took into consideration that people should vaccinate their children against measles not only on National Vaccination Campaign Days, but also at any time during the year, and *on time*, without delay, since this is the official recommendation. It took into account the vaccination schedule proposed at the time: the age for measles vaccination was changed in 2006, such that the first dose must now be administered when the child is 12 months old,^12^ instead of the previous age of 9-11 months. Delays in administering one vaccine may result in failure to adhere to other immunizations and to complete the vaccination calendar.^13^

### Vaccination scheme in Brazil

Major national Multivaccination Campaign Days are organized every year in Brazil. Their aim is mainly to stimulate people to complete the vaccination schedules determined by federal or state government health agencies. On these occasions, the public are encouraged to take their children to primary care centers to have them vaccinated against polio, independent of their previous vaccination history, and to complete their vaccination schedule, receiving other vaccines to protect against other diseases. Since 1977, a National Vaccination Certificate (the vaccination “card”) has provided an official record of immunization. The vaccinations are all registered on the vaccination card in chronological order, so that the healthcare staff can identify any missing vaccinations. For instance, at the time of planning this study, 10-month-old children who had not received a measles vaccine would be given a vaccination against this disease when the MMR triple viral vaccination (protecting against measles, mumps and rubella) had not been registered.

In Brazil, parents must take children to vaccination clinics (private or public) seven times before they complete five years of age. At these visits, each child receives 19 vaccine doses: 14 shots and five Sabin (oral poliovirus) vaccines. At public primary care centers, vaccines are administered free of charge. It is known, however, that separate injections are associated with compliance issues, and therefore combined vaccines, or vaccines that can be coadministered are being proposed.^14,15^ Ramírez Fernández et al. have stated that the introduction of a new vaccine in a national vaccination calendar can cause people to refuse to have it administered because of the new shot.^16^

In the 1990s, two measles vaccine doses were adopted in Brazil: one when the child reached nine months of age, which could be taken until the age of 11 months according to Health Ministry advice, and a second dose in the fifteenth month, which was meant to increase seroconversion. Starting in 1992, this second shot was given as part of the combined measles, mumps and rubella vaccine (MMR).^17–20^ Recently the vaccination calendar was changed, indicating that the first dose should be taken at the age of 12 months.^12^

The first dose of the measles vaccination has been shown to be very important in preventing measles outbreaks.^21^ These doses are available in Brazilian public primary care centers throughout year, from Monday to Friday, and also on the national Multivaccination Campaign Day. Table 1 shows the Brazilian vaccination schedule.

**Table 1.** Brazilian official calendar for vaccinations in 2001^17^

Age	Vaccine
Neonates	tuberculosis and type B hepatitis
1 to 2 months	diphtheria, tetanus and pertussis (DPT), polio*, hepatitis B and *Haemophilus influenzae* type B (Hib)
4 months	DPT, polio and Hib
6 months	DPT, Hib and polio
9 months	measles, yellow fever†, hepatitis B
15 months	measles, mumps and rubella and DPT
5 or 6 years	DPT and polio
15 years	diphtheria and tetanus

* oral Sabin vaccine is used for polio;

† restricted to endemic regions.

Vaccination for children thus depend on these visits and, consequently, on the parents’ adherence to the idea of keeping the vaccination card up-to-date at all ages. It depends, theoretically, on the parents’ understanding of the notion of vaccination, acceptance of the important and safety of vaccination, and ability to take their children to clinics.

It is believed both within the National Immunization Program and the World Health Organization that it will be technically difficult to eliminate measles in Brazil without a minimum 95% vaccination coverage.^22^ Indeed, Brazil's National Health Foundation (a Health Ministry organization) found that, before the campaign of the year 2000, more than three million Brazilian children were susceptible to measles.^23–25^ Other Latin American countries were affected by measles outbreaks in 1998 and 1999 because of lack of vaccination against measles. This disease kills one million people worldwide every year.^20,24,25^

This failure in coverage in Brazil happened because almost 60% of Brazilian cities were unable to reach 95% measles vaccination coverage in 1999. Furthermore, the measles vaccine does not provide total protection: when shots are given between the ages of nine months and one year, they protect only 80 to 85% of children,^23–25^ or even less.^26^ The best protection (as measured by seroconversion) is achieved between the ages of 12 and 15 months, when it reaches up to 95 to 98%.^2^ After one year of age, vaccine failure can affect 7 to 8% of vaccinated children.^4,27,28^

## OBJECTIVE

The aim of this study was to assess the role of communication channels and other risk factors associated with failure to receive the measles vaccine at the right time in the city of São Paulo, Brazil, up to the year 2000.

## METHOD

### Case-control study

We compared people who vaccinated their children correctly against measles (controls) with those who did not vaccinate their children, or delayed the visit to the primary care center to get the shots, or took them too early, according to the vaccination schedule. Assuming that children who had measles during the great 1997 epidemic in São Paulo^28^ would probably have had an incomplete vaccination card, we searched for the cases mainly in a State Health Bureau database that contained names and addresses of children who had suffered from measles during the great 1997 outbreak. The sample was obtained based on differences of proportions (40% among exposed and 20% among nonexposed individuals), with alpha of 5% and test power (1-beta) of 80%. The neighborhoods were randomly chosen from this database until 61 subjects and 61 controls were interviewed (accordingly to a first-classification scheme: “CL I”), with controls living in the same street or block as the paired-subject. All vaccination cards were checked, so that we could obtain the exact measles vaccination status of each child.

We often found that the child who had suffered from measles during the 1997 epidemic, and who we were looking for as a “no-vaccination case”, had moved and thus this specific case of measles was lost. When this happened, we contacted the next-door neighbor in an attempt to find another unvaccinated child, since this was a study about vaccination compliance. Through this, we often found a new “no-vaccination” case (as expected, since the measles epidemic spread in those areas we visited). This prompted us to search for controls in the same street or block, to be paired with each “no-vaccination” case found.

The first classification (CL I) presumed that this group should have had measles vaccination between the exact date when the child completed 9 months and the date of reaching 11 months of age (for the first dose), and at 15 months of age (for the second). Vaccinations administered not more than 10 days (one week plus one weekend) before the start day or after the end date of the period were deemed to be adequate. The age of 11 months was the limit imposed by the local clinics at that time, for administering the first dose of the measles vaccine to the children. If either of the two doses was missing or was taken more than 10 days away from the correct date, the child was taken to be a “vaccination delay subject” for the purposes of this study.

### Exploratory study

After registering all the data on the 61 pairs, we tried to determine how this population would be divided into two comparison groups if the tolerance period for the vaccination dates were to be varied. To investigate this possibility, we changed the group classification criteria. The second classification (CL II) accepted ± 15 days away from the correct vaccination date. The third classification (CL III) tolerated an even larger period, of ± 20 days. On the other hand, a fourth scheme (CL IV) considered only ± 2 days (i.e. no more than a weekend) of tolerance. Table 2 shows an example of a group pair according to the four classifications.

**Table 2.** Two hypothetical examples of pairs of subjects accordingly to four different classifications

Age		Tolerance			
At first dose	At second dose	10 days	15 days	20 days	2 days
9 m (exact)	No second dose	Subject A	Subject	Subject	Subject
9 m 2 days	14 m 29 days	Control A	Control	Control	Control
8 m 25 days	15 m 20 days	Subject B	Subject	Control	Subject
9 m 2 days	14 m 20 days	Control B	Control	Control	Subject

### Variables and interview

A specifically designed questionnaire, including questions concerning demographic, psychological, cultural, communication and infrastructure variables, was applied at an interview with the child's mother or caregiver. This interview aimed to evaluate her/his knowledge about measles (its symptoms, severity and transmission) and about the vaccine (its existence, doses and age of application), and his/her personal feelings about the vaccination. It also evaluated the communication channels through which people had obtained all the information they had. Table 3 presents the groups of variables studied.

**Table 3.** Groups of variables included in the questionnaire designed for the study. Questions were posed to the child's mother or caregiver

Socioeconomic and educational	Educational level, state from which the family originated, marital status, profession
Knowledge about measles and the vaccine	Concepts about the disease and about the power of protection of the vaccine
Communication channels	Where did she/he get the information about the disease and the vaccine
Feelings	Fear of the disease, fear of shots or feeling sorry, fear of side reactions caused by the vaccines
Opinions about the health service	Trust in the healthcare system, staff and materials used, availability of vaccines and location of the clinic (far or near home)
Vaccination	Vaccination date: during vaccination campaign or at the correct age, opinions about vaccination services and campaigns

The socioeconomic factors considered in the questionnaire were possible difficulties in getting to the vaccination centers (such as lack of transportation) or problems with the vaccine itself (such as lack of vaccines in the center). The psychological and cultural factors included fear of vaccination or receiving shots, fear of vaccine reactions (“my child will get a fever if I vaccinate him/her”) or disbelief in the vaccine (“it doesn't work”). The communication factors studied related to lack of information (“when should I vaccinate?”) or the use of communication tools that were not correctly designed to improve adherence.

Two interviewers (graduates in journalism and in psychology) were trained to ask questions in a homogeneous manner (voice, facial expression and body posture) and the first 20 interviews were performed with both interviewers present. At the end of each day, the answers were checked and inserted in the database.

The interviews were carried out between December 1999 and June 2000.

The procedures were conducted in accordance with the ethical standards of the committee responsible for human experimentation and with the Helsinki Declaration of 1975, as revised in 1983, and were approved by the institution's ethics committee (Faculdade de Medicina da Universidade de São Paulo).

## RESULTS

### Cases and controls andtheir vaccinations

The first classification (CL I) resulted in 61 “vaccination delay subjects” and 61 controls. The latter had their children vaccinated on time or within a 10-day tolerance period from the determined day. When the second classification (CL II) was applied, with 15 days of tolerance, it resulted in 54 “vaccination delay subjects” and 68 controls (the number of controls increased because of the increased tolerance). In the third classification (CL III), with 20 days of tolerance, there were 47 “vaccination delay subjects” and 75 controls. However, when we considered those who had their children vaccinated on time or with just two days of tolerance (i.e. not more than a weekend; CL IV), we found 100 “vaccination delay subjects” and 22 controls. In this series, only three children had not taken any of the doses.

These findings show that, in the sample studied, although people did get their children vaccinated, they rarely did so at the time prescribed by the health agencies. Twenty-four children took the first dose on the wrong dates and 80 took the second measles vaccine dose on the wrong dates (using CL IV).

### Socioeconomic characteristics and opinions about the health service

No demographic variable was related to whether the children were vaccinated correctly or not, regardless of the classification (CL) scheme. The demographic variables studied were the caregiver's age and education level, district where the person was living, state from which the person originated and marital status. Even the caregiver's education level did not show any association with vaccinating correctly against measles.

According to our results, the population did not have any difficulty in transportation to the clinic. This showed that the clinics were well distributed across the city. Our population was prepared to wait for more than 30 minutes to get the shot. However, most of these individuals (98) were concerned about not being able to get the immunization once at the care center, because of lack of vaccines.

Being a housekeeper, instead of having a profession, did not help mothers to take their children to the clinics to vaccinate them (assuming that these people would be more time available for this), regardless of the level of tolerance for the vaccination date that was allowed in this study. On the contrary, housekeepers seemed to delay their children's protection (p = 0.008) for more then 10 days. Neither did the fathers help in this respect: in fact, families composed of mother and father living together (103) did not vaccinate their children on time, but tended to delay or anticipate the shots by 20 days (p = 0.008), when compared with mothers raising their children alone (single or divorced).

### Feelings

Feeling sorry for children who were scheduled to receive shots was associated with a delay in receiving the measles vaccine of more than 10 days (Table 4, p = 0.04).

**Table 4.** Feeling sorry for their children who were scheduled to have injections (a) and not letting this influence the decision on whether or not to correctly vaccinate the children (b), according to differences in the delay tolerance

	Tolerance											
	10 days			15 days			20 days			2 days		
	a	b	p	a	b	p	a	b	p	a	b	p
Subjects	38	23	0.04*	35	19	0.02†	32	15	0.009‡	54	46	0.7§
Controls	27	34		30	38		33	42		11	11	

* Odds ratio = 2.08 and *χ*^2^ = 3.98;

† odds ratio = 2.33 and *χ*^2^ = 5.18;

‡ odds ratio = 2.72 and *χ*^2^ = 6.73;

§ odds ratio = 1.17 and *χ*^2^ = 0.12.

As the tolerance regarding the vaccination date was increased in the exploratory analysis of the data, feeling sorry for children taking injections still had an influence on the decision regarding vaccination (p = 0.02 for 15 days; p = 0.009 for 20 days), as showed in Table 4.

### Knowledge about measlesand the vaccine

About 20% of the people interviewed did not know what measles was (24 caregivers), and 69.6% (85) simply said, “It is a disease”. Among the caregivers of children presenting delays on their vaccination cards, 40 (47%) knew of the disease, and among the controls, 45 (53%) responded correctly, but there was no significant difference between cases and controls with regard to this perception. The remainder had a good notion of its transmissible nature (83%), but only 17% (31) said spontaneously that it is a severe disease. It was only when asked whether measles can or cannot kill a child (when the concept of severity was present) that 109 people agreed that it could (89%). Most of the people cited fever (85 or 69.5%) and exanthema (84 or 69%) as symptoms of measles, but many other symptoms were attributed to measles, such as hair loss, diarrhea etc.

Most of the interviewees (114) accepted that vaccination against measles has protective power and the measles vaccine was recognized by 119. However, 25 people said, “There is no mumps vaccine” (the second dose of measles vaccine is taken together with mumps and rubella vaccines in Brazil, in the form of the MMR vaccine).

### Communication channels

We asked the interviewee to remember how he/she was informed about the vaccination. Only five out of the 122 subjects could not remember, and 98 among the remaining 117 (83.7%) said that the information came to them through the television. Television was the only source of information for 62 persons. Radio reached 18 (15.3%).

Nurses and pediatricians customarily mark the next vaccination visit date in the child's vaccination card, using a pencil, as a reminder. Forty-one percent of the people interviewed (51) relied on the health staff to determine when they should get their children vaccinated against measles, by checking this date on the card. No specific communication channel was associated with vaccination compliance.

On the reverse side of the card cover, there is a vaccination calendar with the ages at which each vaccine must be taken. Part of the population (52.42%) usually checked this calendar to see whether it was time to get their children vaccinated. Only 21 people said that they waited until the national Vaccination Campaign Day to get their children vaccinated.

### Indicator for the trendto skip vaccination

Since the answers alone did not discriminate the cases from the controls, they did not explain why people were not having their children vaccinated on time. Therefore, we created an indicator composed of three variables that had the aim of combining the concerns about taking a sick child to be vaccinated or believing that a child with flu, diarrhea or fever could not receive vaccines. The questions that led to the construction of these variables were:

1. If your son/daughter has flu when the vaccination day comes, would you take him/her to the vaccination center to get the shot?
2. If your son/daughter has diarrhea when the vaccination day comes, would you take him/her to the vaccination center to get the shot?
3. If your son/daughter has fever when the vaccination day comes, would you take him/her to the vaccination center to get the shot?

When applied to the sample, the reliability of this “not-to-vaccinate” indicator was tested and verified by means of Cronbach's test (alpha = 0.80). In spite of this consistency, this indicator did not succeed in showing any differences between the two groups.

## DISCUSSION

Two different communication channels for the vaccination schedule stood out from these results as the ones most frequently used: one was the printed calendar on the cards, which showed when it was the time to get the shots. The other was the health staff, through the vaccination card, when the health professionals marked the card with the date for the next visit, which can be called an interpersonal communication. Recently, the Summit of Independent European Vaccination Experts identified that healthcare professionals are “the main advocates for vaccination and the most important source of information about vaccines for the general public” in Europe.^29^ A study in Belgium also showed that vaccinating physicians were a predictor for children to have received all the recommended vaccine doses (complete schedule).^30^

We were informed by the healthcare professionals in the clinics that, until 2002, no specific communication training was supplied to healthcare staff in Brazil with regard to guidance about when and how to provide interpersonal information. This could explain why these channels were inefficient in relation to increasing the vaccination status (or diminishing the delay) among the children studied: neither cases nor controls were significantly associated with any of the channels used, even when mass communication channels like television were considered.

Virtually all the subjects interviewed knew about the 1999 National Vaccination Campaign through television (98) or radio (18), and 62 (50.8%) only through television. Lasso et al. found a similar reach rate in a study carried out in Colombia: 56% of the people got to know about vaccination campaigns through television, radio or the press in general.^31^ The communications about the special vaccination day were effective, comprehensible and reliable for most of the people in our study. But despite the power of its reach among our population, television did not show any ability to change people's behavior regarding measles vaccination specifically, since only 22 children were vaccinated on the strictly correct date.

McDivitt et al., on the other hand, found a different result. In a study carried out in the Philippines in 1990, they showed that a mass communication campaign could promote behavioral change.^32^ These and other authors suggested that two channels in combination — mass and interpersonal — work better than one alone.^32,33^ Almost half (49.2%) of our population knew about the last campaign through channels *other* than television.

Nevertheless, in Brazil, other than the National Vaccination Campaign, there was no systematically planned communication regarding vaccinations during the remainder of the year, according to the healthcare professionals during our investigations. No communication channel was officially used or planned to deliver information about the diseases, or about vaccines on a regular basis, every day. While the vaccination cards could be considered to be a good channel, only 42% of our interviewed sample relied on this channel to seek information about the correct time to vaccinate.

Communication about diseases and vaccines only takes place when health staff talk to families.^9,10,34^ According to our study, individuals believed and relied on the public health staff and on the material used, and 76 (62%) said that they worried about vaccine reactions.

Education, in this study, was not associated with getting vaccinations on time, although other studies had shown that educated people tend to get more vaccines.^7,35–37^

The only significant difference found between the cases and controls in our study was not a structural, economic or communication factor, but a psychological one. People delayed their children's vaccinations out of anxiety: feeling sorry for their children taking injections. As already pointed out here, some studies have shown that the combination of two or more vaccines in a single injection can improve vaccination coverage.^14–16^ As shown in our study, this can really influence the parents/caregivers’ decision whether to take their children to the clinic or not. Most of the people who were anxious about injections delayed the measles vaccination (p = 0.04). To our knowledge, our study was the first to directly address this topic in an interview with caregivers who had not vaccinated their children on time. This psychological variable should be considered in future studies on the role of communication in vaccination compliance, as a possible confounding variable.

One limitation that could possibly be pointed out regarding this study was that the choice of subjects for the study might influence the result concerning knowledge about the disease. The sampling strategy was based on the premise that the children who had had measles in the 1997 outbreak in São Paulo might have been unprotected, i.e. unvaccinated or presenting delayed vaccination (as was indeed proven to be the case when we checked the vaccination cards of the children who had had the disease). This strategy would ensure a randomized search for cases in the areas of the city where the epidemic had spread and where it would be easy to find unvaccinated children, or children with delayed vaccination. Thus, we reached people with delayed vaccination and those who had also had measles. This could have brought in sample bias, since individuals who had suffered from measles obviously would know the disease better. However, because the epidemic occurred in 1997 and our search began in 1999-2000, most of the addresses of measles cases were wrong (because families had moved) and we found more “no-vaccination” cases by chance than through indications from the measles database. Thus, the likelihood that a mother/caregiver in our sample would know about the disease because his/her child had suffered from measles was very low. Moreover, the variable “feeling sorry for their children who were scheduled to have injections” was not associated with any other factors linked to the condition of having had measles or not.

It is possible to speculate that differences in socioeconomic status between cases and controls could have interfered with the results. In fact, we did not evaluate the income of the interviewees. Although we cannot be sure that cases and controls had the same economic power, we can suggest that they had similar family incomes, based on the fact that cases and controls lived in the same neighborhoods, and no other demographic variable, such as access to formal education, was able to differentiate between them. In fact, Anand and Bärnighausen recently showed that “the level of income does not contribute towards improved immunization coverage”,^38^ while Theeten et al. found that employment situation and family income were predictors for a complete schedule.^30^

## CONCLUSION

Many different communication channels are used during the year to inform people about vaccination: not only mass communication, but also the vaccination card itself. Television could potentially reach a larger number of people but it did not necessarily convince them to vaccinate, as shown in this study. Our results suggest that two or more channels should be considered, and a communication plan operating throughout the year should emphasize the correct dates for vaccination, in order to avoid delays in health care center visits to take vaccinations. Feeling sorry for children when receiving shots is associated with a delay in taking the vaccine.
